# Injuries of the Head of the Fœtus

**Published:** 1834

**Authors:** 


2. *Injuries of the head of the Fœtus in passing through the Pelvis.*—Mr. Bradford one of the surgeons of the Manchester Lying-in Hospital, has a paper on this subject (*Lon. Med. and Surg. Jour.*) from which we make the following extracts, as worthy the attention of our practical readers:

"Children, whose heads have suffered pressure in the direction of the occipito-frontal diameter, are frequently born dead, or they die soon after birth, unless the case is properly considered. They are unable to effectually commence the important functions of respiration. The lungs are only partially filled with air by the convulsive sobs which take place. The action of the heart is not free; and if the pulsations in the funis continue, they are labored and oppressed. The countenance is turgid, and of a livid color, and the vessels of the conjunctiva are quite injected with blood. These symptoms fully prove, that the difficulty to commence respiration does not depend upon a mechanical obstruction from mucus in the trachea, but on the injury which the brain has suffered. This organ is in a state of apoplexy, sometimes depending on a very highly congested state of the bloodvessels alone, sometimes on an effusion of blood which takes place in different parts of the brain, and also varies in quantity."

"The pressure applied to the fore and hind part of the head, has a tendency to change the relative situation of many parts of the brain. It forces one hemisphere from the other, which if carried beyond a certain degree, will inevitably produce laceration of the coats of the veins, which pass to the longitudinal sinus; and this danger is increased by the great congestion which exists.

The practice to be adopted, is to bleed freely as soon as possible after the child is born. The funis ought to be divided before it has ceased to pulsate, as no blood can be obtained if this be neglected. If the pulsations in the vessels of the funis have ceased, two leeches must be applied to the temples.

If these means have not been adopted, convulsions generally ensue."

